# Provincial lessons on long-term care implementation in Thailand: motivation and managerial capacities within a decentralized health system in a middle-income country

**DOI:** 10.1186/s12913-026-14571-6

**Published:** 2026-04-18

**Authors:** Thanwarat Thongngun, Sariyamon Tiraphat

**Affiliations:** Department of Public Health Administration, Faculty of Public Health, Mahidol University, 420/1 Ratchawithi Road, Ratchathewi, Bangkok, 10400 Thailand

**Keywords:** Long-term care, Managerial capacity, Workforce motivation, Decentralization, Health systems strengthening, Low- and middle-income countries

## Abstract

**Background:**

Low- and middle-income countries (LMICs) face accelerating demand for long-term care (LTC) amid rapid population ageing, yet system readiness and managerial capacity remain uneven. Thailand’s decentralized LTC model relies heavily on local administrative organizations and frontline managers, underscoring the multifaceted managerial factors involved in translating policy into consistent implementation. However, empirical evidence identifying the specific determinants of implementation performance remains limited. This study examines the contributions of managerial processes, organizational capacity, intrinsic motivation, and work stress to LTC implementation performance. The findings offer policy-relevant insights to strengthen LTC systems in Thailand and other resource-constrained LMIC settings.

**Methods:**

A province-wide cross-sectional survey was conducted among all 131 LTC system managers in Prachuap Khiri Khan, Thailand. A validated instrument assessed administrative management (Drucker’s 4M and Fayol’s POCCC frameworks), motivation, supportive factors, and work stress. Ordinal logistic regression (three hierarchical models) identified determinants of LTC implementation. Model fit was evaluated using Deviance and Pearson goodness-of-fit tests.

**Results:**

Motivation and administrative management emerged as significant predictors of LTC implementation, with AORs of 7.94 (95% CI 1.92-32.78) and 5.19 (95% CI 1.34-20.05), respectively, highlighting the centrality of human and managerial capacities. Within the 4M framework, only the Management domain remained statistically significant (AOR= 6.37, 95%CI 1.96-20.65), whereas manpower and material resources demonstrated no predictive value. Process-level analysis further identified Planning (AOR=5.47, 95%CI 1.06-28.28) and adaptive, moderate-level Controlling (AOR=3.97, 95%CI 1.13-13.90) as statistically significant managerial levers associated with higher implementation performance, reinforcing the pivotal role of targeted managerial functions in driving system-level outcomes.

**Conclusion:**

Findings demonstrate that strengthening LTC in decentralized LMIC systems requires focusing on managerial capability, intrinsic motivation, and adaptive governance—not merely expanding inputs or workforce numbers. Integrating 4M, POCCC, and motivational theory, this study presents a theory-informed implementation model that identifies planning quality and adaptive control as high-impact system levers. Policymakers should prioritize capacity-building architectures, performance learning systems, and participatory planning structures to enhance LTC governance and scalability. Thailand’s experience offers transferable insights for LMICs seeking to advance person-centered, community-based LTC amid fiscal and workforce constraints.

**Supplementary Information:**

The online version contains supplementary material available at 10.1186/s12913-026-14571-6.

## Introduction

Population ageing represents one of the most profound demographic transformations of the 21st century. The number of people aged 65 and older worldwide is projected to increase from 761 million in 2021 to 1.6 billion by 2050, nearly doubling over the next three decades [1]. The fastest expansion is occurring in low- and middle-income countries (LMICs), particularly in East and Southeast Asia, where health and social care systems remain less prepared to meet the complex needs of rapidly ageing populations [2, 3]. In these contexts, the growing prevalence of chronic illnesses, disability, and dependency among older adults has intensified demand for long-term care (LTC). Without robust LTC systems, LMICs risk exacerbating health inequities and overburdening families and primary care services. The responsibility for dependent older persons often shifts from formal institutions to informal household caregivers with limited resources [2]. Consequently, primary-care providers must assume extended roles in chronic and palliative care, diverting scarce resources from preventive and promotive functions [4]. While global initiatives such as the Decade of Healthy Ageing 2021–2030 advocate community-based, integrated LTC frameworks [3], empirical evidence on the determinants of effective LTC implementation in resource-constrained settings remains limited, underscoring the need for more context-specific studies to inform equitable policy development.

Thailand exemplifies this demographic and health-system challenge. The country formally entered an ageing society in the mid-2000s and has continued to experience a steady rise in the proportion of older adults [5]. National reports indicate that a growing share of older people now require assistance with daily living as chronic conditions, physical frailty, and psychosocial vulnerability become more prevalent [6]. These trends reflect a broader epidemiological shift in which non-communicable diseases, functional decline, and dependency increasingly define the health profile of older Thais. Projections suggest that Thailand will become one of the world’s super-aged societies within the next two decades, placing new demands on its health and social care systems [5]. These patterns highlight the need to strengthen comprehensive long-term care (LTC) systems that integrate medical, rehabilitative, and social support within community and home-based settings.

Thailand introduced a public long-term care (LTC) scheme in 2016, in response to these growing needs, through collaboration between the Ministry of Public Health (MoPH) and the National Health Security Office (NHSO) [7, 8]. The Public Health Long-Term Care Fund for Dependent Older Adults is integrated into the Universal Health Coverage (UHC) system to ensure coordinated health and social services for older adults with functional dependency. The program promotes community-based care through health professionals and trained volunteers, who deliver rehabilitation, home visits, and social support activities in partnership with local administrative organizations (LAOs). Under Thailand’s LTC program, care management is guided by national operational guidelines that define the key stages of assessment, care planning, implementation, monitoring, and follow-up. These processes have designated “care managers,” primarily nurses and public health professionals, who serve as the key link between families, community networks, and local governments [6, 9].

Over the past decade, Thailand has built a comprehensive long-term care (LTC) infrastructure integrating policy design, financing mechanisms, and multisectoral governance among the Ministry of Public Health (MoPH), the National Health Security Office (NHSO), and local administrative organizations. Rooted in broader health-sector decentralization reforms, these initiatives have progressively shifted responsibility for LTC planning and service delivery to subnational levels, empowering local administrative organizations (LAOs) and primary care networks to co-manage care for dependent older adults. Such decentralization aims to strengthen local health systems and promote sustainable and equitable service delivery nationwide [10, 11]. Evidence indicates that Thailand’s LTC development has increasingly relied on home- and community-based models, involving collaboration among LAOs, community volunteers, and families as frontline caregivers [12, 13]. Nevertheless, persistent constraints—such as limited managerial readiness, workforce shortages, and weak coordination between health and social sectors—continue to challenge effective implementation [32, 33, 43].

In this context, regional disparities in long-term care (LTC) performance remain substantial. In Health Region 5, which comprises eight provinces, variations in system readiness and service delivery outcomes are particularly pronounced. Prachuap Khiri Khan, in particular, faces disproportionately greater implementation challenges, as reflected in a higher proportion of dependent older adults, lower performance in care planning, and a smaller proportion of subdistricts meeting service standards [14]. These patterns suggest that, beyond structural availability, systemic constraints continue to hinder the effective translation of policy into practice at the local level. While prior research in Prachuap Khiri Khan has provided important initial insights—particularly identifying administrative management as a key determinant of LTC operations [14]—existing evidence remains limited in scope and analytical depth, largely focusing on aggregate-level associations without adequately elucidating underlying managerial processes or the interaction between organizational capacity and human factors. Consequently, variations in implementation within decentralized systems remain insufficiently understood. To address this gap, this study extends prior evidence by explicitly modelling the interplay between managerial capacity, intrinsic motivation, and work stress within a theory-driven framework, thereby offering a more nuanced, process-oriented understanding of LTC implementation in decentralized health systems.

Understanding the determinants of long-term care (LTC) implementation requires integrating organizational and psychological perspectives. At the organizational level, Drucker’s 4 M framework—encompassing manpower, money, materials, and management—provides a foundation for effective resource stewardship and utilization within health systems [15]. Building upon this resource-based view, Fayol’s POCCC model (planning, organizing, commanding, coordinating, and controlling) elaborates how resources are transformed into coordinated, accountable actions [16]. Complementing these organizational perspectives, psychological theories highlight the human dimension of administration. Herzberg’s Two-Factor Theory of Motivation distinguishes between motivational (intrinsic) factors—including achievement, recognition, work characteristics, responsibility, and career advancement—and supportive-system (extrinsic) factors relating to peer relationships, job stability, personal life, professional status, and compensation [17]. Cooper and Marshall’s occupational stress model further emphasizes how excessive job strain—arising from workload, role conflict, or poor organizational climate—can undermine performance and well-being [18]. Together, these frameworks establish an integrated theoretical foundation linking administrative capacity, motivation, and stress to LTC implementation within decentralized health systems.

Despite Thailand’s progress and a growing international literature on population ageing, empirical evidence on the determinants of long-term care (LTC) operations within Thailand’s health system remains limited. Much of the Thai literature has focused on demographic change and the well-being of older persons [19, 20], or on population-based indicators of successful and active ageing, rather than on managerial and organizational determinants [21]. In contrast, comparative work on decentralized health systems has underscored how individual, managerial, motivational, and stress-related factors influence implementation outcomes—an area that has seldom been explored in Thailand’s LTC context [10]. Addressing this knowledge gap, the present study investigates determinants of LTC implementation across five domains—individual characteristics, administrative practices, motivation, supportive resources, and work stress—within Thailand’s public health system. Generating locally grounded evidence identifies modifiable factors to strengthen LTC management and offers transferable lessons for other LMICs facing similar demographic transitions and capacity constraints.

## Methods

### Study design

This study employed a cross-sectional analytical design using a structured questionnaire to collect quantitative data. Data were collected from January 3 to January 17, 2024. The questionnaire was developed based on theoretical and managerial frameworks relevant to long-term care (LTC) administration. Its content validity was evaluated by a panel of three experts in public health administration and LTC management. Each expert rated the relevance of every item on a four-point scale, resulting in item-level content validity indices (I-CVI) ranging from 0.80 to 1.00 and an average scale-level CVI (S-CVI/Ave) of 0.98, indicating excellent content validity. Internal consistency reliability was also high across all domains, with Cronbach’s alpha coefficients ranging from 0.89 to 0.98 (see Supplementary Table 1), confirming the instrument’s reliability for field application.

### Setting

The study was conducted in Prachuap Khiri Khan Province, Thailand, where the Ministry of Public Health, in collaboration with local administrative organizations, has implemented community-based long-term care (LTC) services for dependent older adults under the national LTC policy framework.

### Participants

The study population comprised all 131 health system managers responsible for administering and coordinating LTC operations across the entire province. Given the relatively small number of LTC managers, a census approach—covering all eligible managers in the province—was adopted rather than random sampling to ensure representativeness and strengthen the study’s analytical power, despite the small population. Eligible participants were required to (1) have at least six months of work experience in LTC management within the province and (2) provide voluntary informed consent before participation. Ethical approval was obtained from the Public Health Human Research Ethics Committee of the authors’ institute (Approval No. MUPH 2023 − 143; Project Code 155/2023; Date of approval: 20 November 2023).

### Research instrument

The research instrument was a structured questionnaire consisting of six sections. The questionnaire was developed through a literature review, drawing on established administrative and psychological theories and on adaptations from validated instruments used in previous studies. The core questionnaire had been previously developed and applied in a provincial-level study among public health long-term care system managers in Thailand [14]. The present study employs the same core instrument while extending its application to a broader analytical framework with additional explanatory variables.

#### Section 1: Personal characteristics

This section collected demographic information, including sex, age, marital status, education level, monthly income, and self-rated physical health status.

#### Section 2: Administrative management of long-term care (32 items)

Items were developed based on *Drucker’s 4 M framework*—manpower, money, materials, and management [15], and *Fayol’s POCCC model* (planning, organizing, commanding, coordinating, and controlling) [16]. The content was further adapted from Nokhunthod [22] and Kongsamrit [23], encompassing four domains: human resources, budget, materials and equipment, and administrative processes (planning, organizing, commanding, coordinating, and controlling). Each item was rated using a five-point Likert scale ranging from 1 (“very low”) to 5 (“very high”).

#### Section 3: Motivation for LTC implementation (30 items)

This section was constructed using *Herzberg’s Two-Factor Theory* [17] and adapted from Noimanee [24]. It comprised two domains:

(1) Motivational (intrinsic) factors **—** achievement, recognition, job characteristics, responsibility, and career advancement (15 items); and.

(2) Supportive-system (extrinsic) factors — peer relations, job stability, personal life balance, professional status, and remuneration (15 items).

Responses were rated on a five-point Likert scale (1 = very low, 5 = very high).

#### Section 4: Work stress (10 items)

Guided by *Cooper and Marshall’s (1976)* occupational stress model [18], and adapted from Thammasathir [25], this section measured perceived work stress across four dimensions: workload, role conflict, lack of support, and organizational climate. Each item was scored on a five-point Likert scale from very low to very high.

#### Section 5: Implementation of LTC operations (21 items)

Developed from the Care Manager Training Manual for the Long-Term Care System for Dependent Older Adults [26], this section assessed seven stages of LTC implementation: screening, assessment, care planning, implementation, monitoring, evaluation, and review. Each was rated on a five-point frequency scale (1 = never, 5 = always).

#### Section 6: Open-ended questions

Two items were included to explore challenges and recommendations regarding LTC operations among provincial LTC managers.

All sections were summed and averaged to produce composite scores. In Sects.  2, 3, and 5, items with higher values indicate stronger perceptions or better performance in each domain. Whereas Sect.  4, which measured work stress, was scored in the reverse direction—higher values indicated greater stress and thus lower desirability. Composite scores were categorized into three levels based on percentile cut-offs: low (P₁–P₃₃), moderate (P₃₄–P₆₇), and high (P₆₈–P₁₀₀), reflecting the relative strength of each construct among respondents.

### Data analysis

Data were analyzed using IBM SPSS Statistics version 29 [27]. Descriptive statistics—including frequencies, percentages, means, standard deviations, and percentiles—were used to summarize participant characteristics and levels of LTC administration, motivation, supportive factors, work stress, and implementation.

Ordinal logistic regression analysis was performed to identify determinants of LTC implementation, with statistical significance established at *p* < 0.05.

Three hierarchical models were sequentially specified to explore the structure of influencing factors:

- Model 1 incorporated all conceptual domains derived from the theoretical framework—personal characteristics (sex, age, education, income, marital status, physical health), motivational factors, supportive factors, work stress, and overall administrative management —to identify global predictors of LTC implementation levels.
- Model 2 decomposed the overall administrative management construct into Drucker’s 4 M components (*Man*,* Money*,* Material*, and *Management*) to examine which resource dimension remained significant after excluding motivational, supportive, and stress variables.
- Model 3 further disaggregated the “Management” component according to Fayol’s POCCC framework (*Planning*,* Organizing*,* Commanding*,* Coordinating*, and *Controlling*) to investigate process-level managerial mechanisms underlying LTC performance.

Motivational, supportive, and stress factors were intentionally excluded from Models 2 and 3 to avoid over-adjustment and to focus on internal managerial processes, while personal covariates were retained as control variables in all models.

Model fit was evaluated using the Deviance goodness-of-fit test; a p-value greater than 0.05 indicated an adequate fit between the model and the observed data [28].

## Results

### Socio-demographic characteristics and physical health condition of long-term care system managers, prachuap khiri khan province

Table 1 presents analytical characteristics of long-term care (LTC) system managers (*N* = 131) who participated in the study. The majority of respondents were female (88.5%), while male participants accounted for 11.5%. The mean age was 41.8 ± 9.17 years (range 27–62), with most respondents aged 25–54 years (90.8%), reflecting a predominantly middle-aged workforce.

In terms of marital status, over half (54.2%) were married or cohabiting, followed by single (35.9%) and widowed/divorced/separated (9.9%). Nearly all respondents held at least a bachelor’s degree (93.9%), and only a small proportion attained a master’s degree or higher (6.1%), indicating a generally well-educated group. Regarding economic status, a majority (60.3%) reported earning more than 30,000 THB per month, while 39.7% earned less. Regarding physical health, most participants rated their health as good (59.5%), whereas 40.5% rated it as poor.

Overall, these findings indicate that the LTC workforce in this study consisted mainly of well-educated, middle-aged, and healthy female professionals with stable socioeconomic backgrounds—reflecting a relatively strong human resource base within the LTC system.

**Table 1:** Analytical characteristics of long-term care (LTC) system managers included in the study (*N* = 131)

Variables	Category	*n*	%
**Demographic profile**			
Sex	Male	15	11.5
	Female	116	88.5
Age (years) Mean ± SD (range) = 41.8 ± 9.17 (27–62)	25–34	41	31.3
	35–44	38	29.0
	45–54	40	30.5
	≥ 55	12	9.2
Marital status	Single	47	35.9
	Divorced/Widowed	13	9.9
	Married	71	54.2
**Socio-economic characteristics**			
Educational attainment	Bachelor’s degree	123	93.9
	Master’s degree or higher	8	6.1
Average monthly income (THB)	≤ 30,000	52	39.7
	> 30,000	79	60.3
**Physical health status**	Poor	53	40.5
	Good	78	59.5

Note. Values are presented as frequencies and percentages unless otherwise specified. Continuous variables are reported as mean ± standard deviation (SD) with range. Categories are ordered to align with the coding scheme used in the ordinal logistic regression models, with the reference group last

### Levels of LTC operation and predictors

Table 2 presents the perceived levels of LTC operation and associated influencing factors among health service providers.

One-third of respondents (36.6%) rated LTC operations as low, while 32.1% rated them high. Motivational factors were reported at moderate (38.9%) or low (37.4%) levels, indicating opportunities to improve individual drive and morale.

Similarly, supportive factors showed a comparable distribution, with 38.2% at the moderate level and 37.4% at the low level. Regarding work stress, responses were evenly distributed across the three levels (36.6%, 31.3%, and 32.1%), suggesting diverse perceptions of stress within the workforce.

For the overall domain of LTC administration, nearly two-thirds of participants perceived performance at medium-to-high levels, with high at 30.5% and medium at 32.8% Decomposing LTC administration by Drucker’s 4 M, the highest proportion of high ratings was observed in Management (32.8%), followed by Money (29.8%), whereas Man (18.3%) and Material (16.8%) exhibited comparatively lower high-level shares.

Within the Management dimension (Fayol’s POCCC), the distribution of *high* ratings ranged from 26.0% to 30.5%, with planning (30.5%) leading, followed by Commanding (28.2%), Coordinating (27.5%), Organizing (26.0%), and Controlling (26.0%). Overall, these patterns indicate a tilt toward higher perceived performance in managerial capability—especially planning—while human and material resources are rated at a high level less frequently.

**Table 2:** Perceived Levels of LTC Operation, Motivation, Support, Stress, and Administrative Subdimension (*n* = 131)

Variable	Low *n* (%)	Medium *n* (%)	High *n* (%)
**1. LTC Operation**	48 (36.6)	41 (31.3)	42 (32.1)
**2. Motivational Factors**	49 (37.4)	51 (38.9)	31 (23.7)
**3. Supportive Factors**	49 (37.4)	50 (38.2)	32 (24.4)
**4. Work Stress**	48 (36.6)	41 (31.3)	42 (32.1)
**5. Overall LTC Administration (4M)**	48 (36.6)	43 (32.8)	40 (30.5)
5.1 Man (Human Resources)	51 (38.9)	56 (42.7)	24 (18.3)
5.2 Money (Budgetary Resources)	49 (37.4)	43 (32.8)	39 (29.8)
5.3 Material (Equipment & Supplies)	46 (35.1)	63 (48.1)	22 (16.8)
5.4 Management (POCCC)	50 (38.2)	38 (29.0)	43 (32.8)
5.4.1 Planning	34 (26.0)	57 (43.5)	40 (30.5)
5.4.2 Organizing	36 (27.5)	61 (46.6)	34 (26.0)
5.4.3 Commanding	32 (24.4)	62 (47.3)	37 (28.2)
5.4.4 Coordinating	26 (19.8)	69 (52.7)	36 (27.5)
5.4.5 Controlling	45 (34.4)	52 (39.7)	34 (26.0)

Note. Numbers indicate the hierarchical order of constructs derived from the conceptual framework (4 M and POCCC). Percentages are based on total respondents (*n* = 131). Each domain was categorized on a three-level ordinal scale (Low = below the 33rd percentile; Medium = 34th–66th percentile; High = 67th percentile and above) - see Supplementary Table 2

### Model 1: Overall predictors of LTC implementation

Table 3 presents the results of the ordinal logistic regression analysis examining the overall determinants of long-term care (LTC) implementation among health service providers. The model demonstrated acceptable fit to the data with Deviance = 198.61, df = 221, *p* > 0.05; Pearson χ² = 209.20, df = 221, *p* > 0.05; AIC = 250.71; BIC = 305.34.

Two predictors showed statistically significant associations with LTC implementation at the 0.05 level. First, motivational factors exerted a strong positive influence: respondents with high perceived motivation were 7.94 times more likely to achieve higher levels of LTC implementation than those with low motivation (AOR = 7.94, 95% CI = 1.92–32.78). Second, overall LTC administration also demonstrated a significant effect: participants working in facilities with strong administrative capacity were 5.19 times more likely to achieve higher implementation levels than those in facilities with low administrative capacity (AOR = 5.19, 95% CI = 1.34–20.05). Other factors—such as sex, education, income, marital status, age, physical health, supportive factors, and work stress—did not reach statistical significance (*p* > 0.05). However, the association with respondents aged 45–54 approached significance (*p* = 0.06), suggesting a possible trend toward better LTC implementation performance among mid-career professionals.

**Table 3:** Ordinal logistic regression model 1: predictors of long-term care (LTC) operation level (1 = low, 2 = medium, 3 = high), Overall predictors: personal covariates, motivational, supportive, stress, and overall management factors

Predictors	Adjusted Odds Ratio (AOR)	95% CI	*p*-value
**Sex**			
Male	1.36	0.41–4.52	0.62
Female (Reference)	1	–	–
**Education**			
Lower than a Bachelor’s	3.33	0.62–17.83	0.16
Bachelor’s or higher (Reference)	1	–	–
**Income (THB/month)**			
≤ 30 000	0.94	0.35–2.49	0.90
> 30 000 (Reference)	1	–	–
**Marital status**			
Single	3.09	0.72–13.39	0.13
Divorced/Widowed	1.40	0.37–5.26	0.62
Married (Reference)	1	–	–
**Age group (years)**			
≤ 30	0.56	0.11–2.77	0.48
31–40	0.56	0.14–2.30	0.42
41–50	4.06	0.92–17.88	0.06†
> 50 (Reference)	1	–	–
**Physical health**			
Poor	0.99	0.44–2.24	0.98
Good (Reference)	1	-	-
**Motivational factors**			
High	7.94	1.92–32.78	0.00*
Medium	1.48	0.46–4.78	0.51
Low (Reference)	1	–	–
**Supportive factors**			
High	0.53	0.14–2.03	0.35
Medium	0.93	0.28–3.12	0.91
Low (Reference)	1	–	–
**Work stress**			
High	1.63	0.65–4.12	0.30
Medium	1.37	0.54–3.49	0.51
Low (Reference)	1	–	–
**LTC administration**			
High	5.19	1.34–20.05	0.02*
Medium	2.81	0.90–8.77	0.08
Low (Reference)	1	–	–

Note. * indicates significance at *p* < 0.05; † indicates marginal significance at *p* < 0.10., Model Fit Statistics:(Deviance = 198.61, df = 221, *p* > 0.05; Pearson χ² = 209.20, df = 221, *p* > 0.05; AIC = 250.71; BIC = 305.34)

### Model 2: Resource-based determinants of LTC implementation (4 M framework)

The ordinal logistic regression model (Model 2), as shown in Table 4, exhibited an acceptable fit to the data. The deviance-to-degree-of-freedom ratio was approximately 1.00 (Deviance = 204.983, df = 205), and the Pearson chi-square/df ratio was 1.016, indicating a well-fitting model without evidence of over- or under-dispersion. The information criteria values (AIC = 256.51, BIC = 311.14) were also satisfactory, indicating that the model balances goodness-of-fit and parsimony.

In Model 2, which decomposed the LTC administrative management construct into Drucker’s 4 M dimensions, only the management domain emerged as a statistically significant determinant of LTC implementation. Specifically, respondents working in facilities with high management performance levels were 6.37 times more likely to report higher LTC implementation levels than those in facilities with low management performance (AOR = 6.37, 95% CI = 1.96–20.65). This finding demonstrates that facilities with strong managerial processes—namely, planning, organizing, commanding, coordinating, and controlling (POCCC)—tend to achieve more effective LTC implementation outcomes.

Budgetary resources (Money) showed a marginally positive association with LTC performance (AOR = 3.12, 95% CI = 0.92–10.60), suggesting that financial sufficiency may enhance LTC operations, although this relationship did not reach statistical significance. Conversely, the human resource (Man) and material and equipment (Material) domains did not exhibit significant effects on LTC implementation (*p* > 0.05).

Demographic covariates—including sex, education, income, marital status, age, and physical health status—were not statistically significant predictors (*p* > 0.05), suggesting that organizational-level management capacity, rather than individual characteristics, plays a more decisive role in determining LTC implementation outcomes.

**Table 4:** Ordinal logistic regression model 2: predictors of LTC operation level – 4 M framework (Man, Money, Material, and Management)

Predictors	Adjusted Odds Ratio (AOR)	95% CI	*p*-value
**Sex**			
Male	1.33	0.40–4.42	0.64
Female (Reference)	1	–	–
**Education**			
Lower than a Bachelor’s	1.66	0.33–8.28	0.54
Bachelor’s or higher (Reference)	1	–	–
**Income (THB/month)**			
≤ 30,000	0.91	0.34–2.46	0.86
> 30,000 (Reference)	1	–	–
**Marital status**			
Single	1.96	0.46–8.31	0.36
Divorced/Widowed	1.26	0.32–4.95	0.74
Married (Reference)	1	–	–
**Age group (years)**			
≤ 30	0.50	0.10–2.45	0.39
31–40	0.36	0.09–1.50	0.16
41–50	2.47	0.60–10.11	0.21
> 50 (Reference)	1	–	–
**Physical health**			
Poor	0.82	0.36–1.85	0.64
Good (Reference)	1	–	–
**Man (Human Resources)**			
High	0.67	0.16–2.79	0.59
Medium	1.00	0.30–3.36	1.00
Low (Reference)	1	–	–
**Money (Budgetary Resources)**			
High	3.12	0.92–10.60	0.07†
Medium	2.40	0.73–7.92	0.15
Low (Reference)	1	–	–
**Material and Equipment**			
High	1.45	0.33–6.35	0.62
Medium	0.90	0.22–3.66	0.88
Low (Reference)	1	–	–
**Management**			
High	**6.37**	**1.96–20.65**	0.00*
Medium	2.15	0.72–6.42	0.17
Low (Reference)	1	–	–

Note. * indicates significance at *p* < 0.05; † indicates marginal significance at *p* < 0.10., Model Fit Statistics: Deviance = 204.983 (df = 205); Deviance/df = 1.00; Pearson χ²/df = 1.016; AIC = 256.51; BIC = 311.14

### Model 3: Process-level determinants of LTC implementation (POCCC framework)

Model 3, as shown in Table 5, further decomposed the management construct into Fayol’s five managerial functions (POCCC Framework)—Planning, Organizing, Commanding, Coordinating, and Controlling—to identify specific process-level determinants of LTC implementation. The model demonstrated an acceptable fit to the data (Deviance/df = 0.90; Pearson χ²/df = 0.93) and achieved the lowest information criteria among all models (AIC = 236.93; BIC = 297.31), confirming its optimal parsimony and explanatory power.

Among the POCCC dimensions, planning and controlling emerged as statistically significant predictors of LTC implementation. Facilities with high levels of planning performance were 5.47 times more likely to achieve higher LTC implementation levels than those with low planning performance (AOR = 5.47, 95% CI = 1.06–28.28).

Within Fayol’s managerial functions, the controlling dimension was statistically significant at the moderate level (AOR = 3.97, 95% CI = 1.13–13.90), indicating that facilities with moderate levels of administrative control were approximately four times more likely to achieve higher LTC implementation levels compared with those with low control (reference group). In contrast, the high-control group showed only marginal significance (AOR = 3.99, 95% CI = 0.87–18.27). These results suggest that a balanced, moderate degree of control may foster better LTC performance than overly strict or lax control mechanisms.

Other managerial functions—including organizing, commanding, and coordinating—were not statistically significant (*p* > 0.05). Demographic covariates (sex, education, income, marital status, age, and physical health) also remained non-significant, underscoring the dominant role of organizational management processes over individual characteristics in explaining LTC implementation outcomes.

**Table 5:** Ordinal logistic regression model 3: predictors of LTC operation level – POCCC framework (Planning, Organizing, Commanding, Coordinating, and Controlling)

Predictors	Adjusted Odds Ratio (AOR)	95% CI	*p*-value
**Sex**			
Male	1.88	0.54–6.53	0.32
Female (Reference)	1	–	–
**Education**			
Lower than a Bachelor’s	2.41	0.45–12.99	0.31
Bachelor’s or higher (Reference)	1	–	–
**Income (THB/month)**			
≤ 30,000	1.00	0.37–2.68	0.99
> 30,000 (Reference)	1	–	–
**Marital status**			
Single	2.19	0.50–9.53	0.30
Divorced/Widowed	1.27	0.33–4.87	0.73
Married (Reference)	1	–	–
**Age group (years)**			
≤ 30	0.40	0.08–2.09	0.28
31–40	0.41	0.10–1.74	0.23
41–50	2.98	0.68–13.10	0.15
> 50 (Reference)	1	–	–
**Physical health**			
Poor	0.71	0.32–1.57	0.40
Good (Reference)	1	–	–
**Planning**			
High	**5.47**	**1.06–28.28**	0.04*
Medium	1.59	0.43–5.86	0.49
Low (Reference)	1	–	–
**Organizing**			
High	0.43	0.07–2.47	0.34
Medium	0.38	0.09–1.64	0.20
Low (Reference)	1	–	–
**Commanding**			
High	4.30	0.57–32.66	0.16
Medium	1.75	0.41–7.43	0.45
Low (Reference)	1	–	–
**Coordinating**			
High	1.30	0.17–9.95	0.80
Medium	1.11	0.23–5.35	0.90
Low (Reference)	1	–	–
**Controlling**			
High	3.99	0.87–18.27	0.08†
Medium	**3.97**	**1.13–13.90**	0.03*
Low (Reference)	1	–	–

Note: * indicates significance at *p* < 0.05; † indicates marginal significance at *p* < 0.10., Model Fit Statistics: Deviance/df = 0.90; Pearson χ²/df = 0.93; AIC = 236.93; BIC = 297.31

## Discussion

This study identified empowerment-oriented motivation and administrative management—particularly the processes of planning and moderate control—as important determinants of long-term care (LTC) implementation among public health-care managers in Thailand’s provincial system. The results suggest that successful LTC delivery for dependent older adults relies not merely on material or financial resources but fundamentally on the human and managerial dimensions that transform institutional frameworks into sustained practice.

Motivation was among the strongest factors associated with the implementation of long-term care (LTC) operations. The study incorporated five subcomponents—achievement, recognition, job characteristics, responsibility, and career advancement—that represent empowerment-based psychological drivers that reinforce intrinsic energy, professional ownership, and commitment among care managers. These findings are consistent with Herzberg’s motivation–hygiene theory, which identifies achievement, recognition, and responsibility as key sources of work satisfaction [29], and with Kanter’s structural empowerment theory, which posits that access to power, opportunity, and information fosters autonomy and persistence in organizational contexts [30]. Within Thailand’s decentralized LTC system—where district health networks, local administrative organizations (LAOs), and the National Health Security Office (NHSO) coordinate services under the Universal Coverage Scheme—empowerment-based motivation may enable managers to sustain service implementation despite limited resources. Similar dynamics have been reported in other low- and middle-income countries (LMICs), where non-monetary intrinsic drivers such as purpose, recognition, and autonomy support long-term workforce performance and program sustainability [31–33]. In East and Southeast Asia, evidence from Japan shows that intrinsic rather than extrinsic motivation predicts work engagement among LTC nurses [34], while the WHO’s *Integrated Care for Older People (ICOPE)* framework emphasizes that social participation and self-care capacity are essential for maintaining intrinsic functioning and motivation among older persons [35–37].

At the organizational level, structured and participatory planning appears to play a more prominent role than the simple expansion of staff or financial resources in effective LTC implementation. Evidence from the SUSTAIN Project (Sustainable Tailored Integrated Care for Older People in Europe) demonstrated that organizations that co-designed and regularly reviewed person-centered improvement plans achieved stronger coordination, continuity, and quality of care than those without systematic planning processes [38]. A complementary analysis of fourteen European integrated-care sites further confirmed that well-structured improvement plans—with clear objectives, milestones, and feedback loops—translated national policy priorities into local operational changes and measurable service gains ([39]. These findings are consistent with the World Health Organization’s Integrated Care for Older People (ICOPE) Implementation Framework, which advocates readiness assessments and action plans at the meso and macro levels to ensure alignment between policy goals and service delivery [40–41]. Reinforcing this, the Royal Commission into Aged Care Quality and Safety in Australia identified weak planning, fragmented governance, and limited accountability as major barriers to safe and person-centered LTC—emphasizing that data-driven planning and continuous review are indispensable for sustainable system performance [42].

Beyond structural inputs, our findings suggest that adaptive managerial control—rather than rigid supervision—may be more strongly associated with effective LTC performance. This pattern reflects a transition from hierarchical oversight to enabling governance, emphasizing participatory review, data-driven feedback, and iterative adaptation. Such mechanisms align closely with the World Health Organization’s Integrated Care for Older People (ICOPE) Implementation Framework, which advocates readiness assessment, flexible management, and continuous quality improvement through multi-level action plans [40, 41]. In Thailand, these adaptive principles are reflected in ongoing governance reforms led by the National Health Security Office (NHSO) and the Department of Health Service Support (DHSS)—including the national LTC digital dashboard, performance-reporting systems for care managers, and district-level review meetings emphasizing collective learning rather than inspection [7, 8]. The Asian Development Bank’s Country Diagnostic Study on Long-Term Care in Thailand further highlights that strengthening decentralized planning, local accountability, and feedback mechanisms remains vital to scaling integrated LTC systems [43]. Consistent with this view, the Royal Commission into Aged Care Quality and Safety in Australia found that fragmented governance, weak planning, and limited oversight undermined the quality of LTC, reinforcing the need for adaptive, feedback-oriented control frameworks [42].

From a policy standpoint, these findings underscore that Thailand’s LTC reform should invest more critically in strengthening managerial and motivational empowerment within its decentralized care system. First, the Ministry of Public Health (MoPH) and the National Health Security Office (NHSO) should institutionalize structured capacity-building programs that nurture achievement, recognition, professional responsibility, and career advancement—key psychological enablers of intrinsic motivation among LTC managers. Second, participatory and data-informed planning mechanisms must be embedded within provincial and district LTC boards to translate national strategies into contextually relevant, community-responsive care plans, thereby reducing fragmentation across local administrative organizations (LAOs), health networks, and hospitals. Third, the government should advance adaptive monitoring and learning systems that integrate service data, budget tracking, and performance feedback—promoting a culture of continuous improvement and mutual accountability rather than punitive inspection. Such governance arrangements would foster an empowered, learning-oriented LTC system capable of sustaining quality and equity amid demographic and fiscal pressures.

The strength of this study lies in its theory-informed analytical design, which integrates Drucker’s 4 M and Fayol’s POCCC frameworks to empirically examine how motivational and managerial capacities influence the implementation of long-term care (LTC) within a provincial health system. As one of the few province-wide, field-based analyses in low- and middle-income countries (LMICs), it provides rare empirical evidence on how local governance and organizational processes can be leveraged to deliver equitable and sustainable LTC aligned with global healthy-ageing and UHC goals. The study thus offers a transferable model for decentralized LTC development applicable not only to Thailand but also to similar health systems in LMICs.

However, several limitations should be acknowledged. First, this study was conducted in a single province, which may limit the generalizability of the findings to other regions of Thailand with different socio-cultural and administrative contexts. Together with variations in local governance capacity and resource environments across provinces, this may result in different implementation dynamics. Additionally, the cross-sectional design limits causal inference regarding the relationships between managerial, motivational, and stress-related factors and LTC implementation outcomes. Building on these findings, future research employing multi-provincial or comparative designs, combined with mixed-methods approaches, would be valuable for validating and extending them for broader policy application. Such approaches could further explore how planning and adaptive control mechanisms influence service quality, user experience, and cost-effectiveness across diverse provincial settings and international contexts, thereby advancing comparative understanding of effective LTC governance.

## Conclusion

This study provides empirical evidence highlighting the important role of empowerment-oriented motivation and administrative management—particularly planning and adaptive control—in shaping long-term care (LTC) implementation within Thailand’s provincial health system. The findings move beyond the conventional resource-based paradigm to demonstrate that system performance in LTC depends not merely on funding or staffing, but on the human and managerial capacities that convert institutional frameworks into continuous and equitable care delivery.

By integrating Drucker’s 4 M and Fayol’s POCCC frameworks, this study contributes, both theoretically and empirically, to understanding how psychological and managerial mechanisms interact to sustain service delivery in decentralized health systems. Motivation—especially empowerment-related components such as achievement, recognition, work responsibility, and advancement—acts as the psychological pathway through which individual agency translates into consistent frontline performance. At the organizational level, planning and adaptive control are systemic levers that align micro-level motivation with macro-level policy objectives, ensuring coherence, feedback, and learning throughout the care continuum.

These findings advance the global discourse on integrated care and align strongly with the World Health Organization’s Integrated Care for Older People (ICOPE) framework, emphasizing flexibility, intersectoral collaboration, and person-centered governance. Thailand’s LTC experience suggests that, even in resource-constrained settings, empowerment-driven motivation and participatory managerial processes can support improvements in coordination and continuity of care. This insight provides potentially transferable lessons for other low- and middle-income countries (LMICs) seeking to institutionalize people-centered, community-based LTC systems under fiscal and workforce limitations.

From a policy perspective, Thailand’s Ministry of Public Health and the National Health Security Office should further institutionalize empowerment-based capacity building, participatory planning, and adaptive monitoring systems, emphasizing learning and accountability rather than compliance. Investing in these “soft infrastructure” domains—human capability, organizational climate, and managerial autonomy—may offer a higher return on system performance than purely financial expansion. At the same time, incorporating empowerment and management indicators into national LTC performance dashboards would help measure progress toward *People Excellence* and *Healthy Ageing* strategies.

Ultimately, the dual pathway revealed here—linking empowerment motivation and managerial capacity—highlights that sustainable LTC implementation is as much a matter of *how people are led and inspired* as it is of *how systems are structured and financed*. In practice, Thailand’s LTC reform offers a living laboratory for LMICs transitioning toward integrated, person-centered care. Embedding empowerment, planning, and adaptive control into the organizational DNA of public health institutions can foster resilience, equity, and sustainability—bringing global aspirations for healthy ageing closer to realization.

## Supplementary Information

Below is the link to the electronic supplementary material.

Supplementary Material 1

## Data Availability

The datasets used and/or analyzed during the current study are not publicly available due to ethical restrictions and the presence of sensitive information, but are available from the corresponding author on reasonable request, subject to approval by the Human Research Ethics Committee, Faculty of Public Health, Mahidol University.

## Abbreviations

4 M: Man, Money, Material, and Management
DHSS: Department of Health Service Support
ICOPE: Integrated Care for Older People
I-CVI: Item-level Content Validity Index
LAOs: Local Administrative Organizations
LMICs: Low- and middle-income countries
LTC: Long-term care
MoPH: Ministry of Public Health
NHSO: National Health Security Office
POCCC: Planning, Organizing, Commanding, Coordinating, and Controlling
SUSTAIN: Sustainable Tailored Integrated Care for Older People in Europe
UHC: Universal Health Coverage
WHO: World Health Organization
