# Supplementary material for: Provincial lessons on long-term care implementation in Thailand: motivation and managerial capacities within a decentralized health system in a middle-income country
Source: BMC Health Serv Res. 2026 Apr 18;26:778. doi: 10.1186/s12913-026-14571-6 (PMC13224624; doi:10.1186/s12913-026-14571-6)
Supplement: Supplementary file 1 — Supplementary Material 1 [file 12913_2026_14571_MOESM1_ESM.docx]

# Supplementary Data

Supplementary Tables 1–3: Reliability, Descriptive Statistics, and Collinearity Statistics for Predictors in Ordinal Logistic Regression Models

Supplementary Table 1. Variables and Reliability of Measurement Domains

| **Domain** | **Number of items** | **Example items** | **Scale** | **Cronbach’s α** |
| --- | --- | --- | --- | --- |
| Motivation | 15 | “I am proud to be a long-term care (LTC) manager.” | 5-point Likert | 0.93 |
| Supportive resources | 15 | “Training on LTC management is provided regularly.” | 5-point Likert | 0.89 |
| Work stress | 10 | “I often feel mentally exhausted from my LTC duties.” | 5-point Likert | 0.94 |
| Administrative factors (4M: Man, Money, Material, Management) | 32 | “The use of LTC funds is efficient and cost-effective.” | 5-point Likert | 0.97 |
| Managerial processes (POCCC: Planning, Organizing, Commanding, Coordinating, Controlling)  Long-term care (LTC) implementation | 20  21 | “Plans for LTC service are regularly reviewed and adjusted.”  “I screen and identify older adults requiring LTC services.” | 5-point Likert  5-point Likert | 0.98  0.97 |

Note: Each domain score was computed as the mean of all items. Higher scores indicate stronger perception or performance, except for work stress, which is reverse-scored.

## Supplementary Table 2. Descriptive Statistics of All Study Variables (n = 131)

| **Variable** | **Mean** | **SD** | **Min** | **Max** | **P33** | **P67** |
| --- | --- | --- | --- | --- | --- | --- |
| 1. LTC Operation | 125.44 | 17.02 | 76.00 | 160.00 | 123.00 | 129.00 |
| 2. Motivational Factors | 14.81 | 2.47 | 7.00 | 20.00 | 14.00 | 16.00 |
| 3. Supportive Factors | 15.68 | 2.45 | 8.00 | 20.00 | 15.00 | 16.00 |
| 4. Work Stress | 14.55 | 2.78 | 7.00 | 20.00 | 13.00 | 16.00 |
| 5. **Overall LTC Administration (4M)** | 80.40 | 11.11 | 43.00 | 100.00 | 79.00 | 81.44 |
| 5.1 Man (Human Resources) | 56.98 | 7.51 | 41.00 | 75.00 | 55.00 | 60.00 |
| 5.2 Money (Budgetary Resources) | 56.65 | 7.25 | 30.00 | 75.00 | 54.00 | 60.00 |
| 5.3 Material (Equipment & Supplies) | 27.98 | 8.05 | 10.00 | 50.00 | 23.56 | 31.44 |
| **5.4 Management (POCCC)** | 93.11 | 12.61 | 54.00 | 105.00 | 91.00 | 102.00 |
| 5.4.1 Planning | 16.31 | 2.41 | 10.00 | 20.00 | 16.00 | 16.00 |
| 5.4.2 Organizing | 15.86 | 2.46 | 8.00 | 20.00 | 16.00 | 16.00 |
| 5.4.3 Commanding | 16.08 | 2.42 | 10.00 | 20.00 | 16.00 | 16.00 |
| 5.4.4 Coordinating | 16.32 | 2.43 | 7.00 | 20.00 | 16.00 | 16.00 |
| 5.4.5 Controlling | 15.83 | 2.52 | 7.00 | 20.00 | 15.00 | 16.00 |

Note: All composite scores were categorized into three levels based on percentile thresholds: Low (P₁–P₃₃), Moderate (P₃₄–P₆₇), and High (P₆₈–P₁₀₀).

**Supplementary Table 3.** **Collinearity Statistics for Predictors**

**in Ordinal Logistic Regression Models (Model 1–3)**

| **Predictor Variable** | Level | Tolerance (Model 1) | VIF (Model 1) | Tolerance (Model 2) | VIF (Model 2) | Tolerance (Model 3) | VIF (Model 3) |
| --- | --- | --- | --- | --- | --- | --- | --- |
| **Motivation** | Moderate | 0.360 | 2.777 | — | — | — | — |
|  | High | 0.307 | 3.254 | — | — | — | — |
| **Supportive resources** | Moderate | 0.440 | 2.273 | — | — | — | — |
|  | High | 0.337 | 2.969 | — | — | — | — |
| **Work stress** | Moderate | 0.664 | 1.506 | — | — | — | — |
|  | High | 0.601 | 1.665 | — | — | — | — |
| **Overall Administration** | Moderate | 0.328 | 3.053 | — | — | — | — |
|  | High | 0.285 | 3.510 | — | — | — | — |
| **Man (4M)** | Moderate | — | — | 0.486 | 2.056 | — | — |
|  | High | — | — | 0.371 | 2.696 | — | — |
| **Money (4M)** | Moderate | — | — | 0.413 | 2.421 | — | — |
|  | High | — | — | 0.338 | 2.958 | — | — |
| **Material (4M)** | Moderate | — | — | 0.497 | 2.012 | — | — |
|  | High | — | — | 0.360 | 2.777 | — | — |
| **Management (4M)** | Moderate | — | — | 0.541 | 1.849 | — | — |
|  | High | — | — | 0.367 | 2.722 | — | — |
| **Planning (POCCC)** | Moderate | — | — | — | — | 0.316 | 3.167 |
|  | High | — | — | — | — | 0.207 | 4.827 |
| **Organizing (POCCC)** | Moderate | — | — | — | — | 0.311 | 3.219 |
|  | High | — | — | — | — | 0.209 | 4.778 |
| **Commanding (POCCC)** | Moderate | — | — | — | — | 0.234 | 4.267 |
|  | High | — | — | — | — | 0.143 | 6.980 |
| **Coordinating (POCCC)** | Moderate | — | — | — | — | 0.262 | 3.824 |
|  | High | — | — | — | — | 0.148 | 6.773 |
| **Controlling (POCCC)** | Moderate | — | — | — | — | 0.342 | 2.925 |
|  | High | — | — | — | — | 0.257 | 3.897 |

**Note:** All predictors—including dummy-coded levels of categorical variables—showed Tolerance > 0.10 and VIF < 10, indicating no serious multicollinearity across Models 1–3. Diagnostics confirmed the independence assumption of the ordinal logistic regression analyses.
